# Association between itching and the serum zinc levels in patients with varicose veins

**DOI:** 10.1186/s40780-017-0092-9

**Published:** 2017-09-21

**Authors:** Yasushi Takai, Keiichi Hiramoto, Yoshiyuki Nishimura, Ryota Uchida, Keigo Nishida, Kazuya Ooi

**Affiliations:** 1 Department of Pharmacy, Mie Heart Center Hospital, 2227-1 Ooyodo, Taki, Meiwa, Mie 515-0302 Japan; 2 Laboratory of Clinical Pharmacology, Graduate School of Pharmaceutical Sciences, Suzuka University of Medical Science, 3500-3 Minamitamagaki, Suzuka, Mie 513-8670 Japan; 3 Laboratory of Immune Regulation, Graduate School of Pharmaceutical Sciences, Suzuka University of Medical Science, 3500-3 Minamitamagaki, Suzuka, Mie 513-8670 Japan; 4 Faculty of Pharmaceutical Sciences, Suzuka University of Medical Science, 3500-3 Minamitamagaki, Suzuka, Mie 513-8670 Japan; 5 Cardiovascular Surgery, Mie Heart Center Hospital, 2227-1 Ooyodo, Taki, Meiwa, Mie 515-0302 Japan

**Keywords:** Zinc, Transepidermal water loss (TEWL), Itching, Stratum corneum water content, Varicose veins

## Abstract

**Background:**

Varicose veins commonly occur in the lower extremities and can cause pain and discomfort in the affected area. Many patients with varicose veins suffer from itching, but its cause has not been sufficiently explained. In recent years, the role of zinc in maintaining the integrity of skin has been reported, and zinc supplementation has been suggested to be effective in relieving itching. The objective of this study is to elucidate the relationship between itching and serum zinc concentration in patients with varicose veins.

**Methods:**

We measured the stratum corneum water content, TEWL and zinc levels in each participant. The study subjects were 11 patients with varicose veins who were experiencing itching (2 males and 9 females, mean age: 65.9 ± 9.4 years old) and 13 patients without itching (6 males and 7 females, mean age: 59.3 ± 9.5 years old). The control group was comprised of 9 healthy individuals without varicose veins (mean age: 41.7 ± 7.0 years old).

**Results:**

The level of stratum corneum water content was significantly lower in the patients experiencing itching compared to those without the symptom, and it was significantly lower in both patient groups than in the control group. Transepidermal water loss (TEWL) was significantly higher in the patients experiencing itching than those without the symptom, and it was significantly higher in both patient groups than in the control group. In addition, zinc level was significantly lower in the patients with itching compared to those without itching or the control group. For all study participants, relationships between the stratum corneum water content and TEWL against serum zinc were examined. There was a positive correlation between the stratum corneum water content and serum zinc, and a negative correlation was found between TEWL and serum zinc.

**Conclusion:**

Our novel findings suggested that the development of varicose veins leads to decrease in serum zinc, causing dry skin which could contribute to itching.

## Background

Development of varicose veins is a common venous disorder in the lower extremities. The rate of occurrence of varicose veins in the adult population varies according to different studies; however, it is evident that it increases with age [1–3]. In addition, it is prevalent among pregnant mothers and those engaged in work that requires prolonged standing [1–4]. Varicose veins are believed to be responsible for a wide range of symptoms such as pain, swelling, and tingling/itching sensation in the affected area [2, 3]. The Edinburgh vein study, a cohort study conducted from 1994 to 1996, showed that up to 19% of male patients and 25.3% of female patients suffer from itchy skin in the area affected by varicose veins [5]. Moreover, it has been reported that patients with atopic dermatitis suffer from itching and skin roughness [6].

Studies in recent years have suggested a link between dermatological problems and zinc deficiency [7]. Zinc is an essential trace element to maintain a healthy body, and its deficiency causes various adverse health effects such as dysgeusia, cacogeusia, delayed or abnormal growth, anemia, anorexia or loss of appetite, delayed wound healing and various skin conditions [8, 9]. Among the major organs, the skin contains zinc at the second highest level following muscles and bones [10]. It is considered that the skin is readily influenced by zinc deficiency, and supplementing zinc to alleviate itching in dialysis patients has been proposed in several reports [11, 12].

Association between serum concentration of zinc and itching in the patients with varicose veins of the lower extremities has yet to be reported. The aim of this study is to elucidate the link between serum zinc levels and itchy skin in the patients with varicose veins.

## Methods

### Patients and control subjects

Patients participated in this study were diagnosed with varicose veins at Mie Heart Center between March 2014 and December 2014. The study patients were evaluated by age, sex, smoking habits, whether they had type II diabetes mellitus or hypertension, family history and birth history. Varicose veins were diagnosed by lower extremity ultrasonography. The control group consisted of healthy volunteers with no medical history. We confirmed that all participants did not consume zinc-containing supplements and zinc-rich foods (e.g oysters, cheese etc.) for at least a few days prior to this study. Written informed consent was obtained from each participant. The protocol of this study was approved by the Mie Heart Center Ethical Review Board.

### Study design

We measured the stratum corneum water content, TEWL and zinc levels in each participant. For the patients experiencing itching, we assessed the intensity of itching by face scale [13]. We received the cooperation of healthy individuals for the controls in all tests performed.

### Itch measurements

Assessment of itching on the skin above varicose veins was performed using a 5-point scale ranging from “0” indicating no itching, to “4” indicating the most intense itching [13].

### Isolation of blood and measurement of zinc content

A 6-ml blood sample was collected from each participant in this study. Plasma was fractionated from collected blood samples by centrifugation at 3000×g for 5 min at 4 °C, and the supernatant fractions were isolated and stored at −80 °C until analysis. The samples were sent to SHINO-TEST corporation (Kanagawa, Japan) for the analysis of zinc content. Zinc content was determined by a of colorimetric method using (ACCURAS AUTO Zn) [14].

### Measurement of stratum corneum water content and TEWL

The level of stratum corneum water content and TEWL were measured using a skin-measuring instrument [Tewameter TM300 and Corneometer CM825 (Courage + Khazaka Electronic GmbH, Cologne, Germany)], applying the measuring probe directly onto the affected area. The measurements were taken at room temperature (between 22 °C and 26 °C) and humidity ranging from 20% to 40%.

### Statistical analyses

Data were compared between patients experiencing itching, patients not experiencing itching and non-patient controls. All data are presented as the mean ± standard deviation, and statistical evaluations were performed using Dunnett’s test or Tukey-Kramer test. A *p*-value of <0.05 was considered statistically significant. Correlation with serum zinc was evaluated using Pearson’s moment correlation coefficient.

## Results

This study included 11 patients experiencing itching (2 males and 9 females, mean age: 65.9 ± 9.4 years old) and 11 patients without such symptom (6 males and 7 females, mean age: 59.3 ± 9.5 years old). The non-patient control group was comprised of 9 individuals without varicose veins (mean age: 41.7 ± 7.0 years old). None of the participants in this study had a family history for varicose veins. All female patients had a history of childbirth. The mean itch score among patients experiencing itching was 1.6 ± 0.9 (Table 1).

**Table 1:** Background of patients and control subjects

Parameter	Study group (itching)		Study group (without itching)		Control group	
	Male	Female	Male	Female	Male	Female
Number of patients, *n*	2	9	6	7	2	7
Age (year;mean ± SD)	75.0 ± 2.8	63.8 ± 9.1	53.5 ± 10.1	64.2 ± 5.7	49.0 ± 0	39.5 ± 7.1
Itch score	2	1.5	0	0	0	0
Smoker,n	2	1	1	0	0	0
Type 2 diabetes mellitus,n	1	1	0	0	0	0
Hypertension,n	0	2	1	1	0	0
Family history of varicosis, n	0	0	0	0	0	0
Birth history (had >1 child),n	0	9	0	7	0	0
Hemoglobin,(g/dL;mean ± SD)	13.2 ± 0.6	12.7 ± 0.8	12.7 ± 0.8	13.1 ± 0.9	14.6 ± 0.6	13.7 ± 0.7

**Fig. 1:**
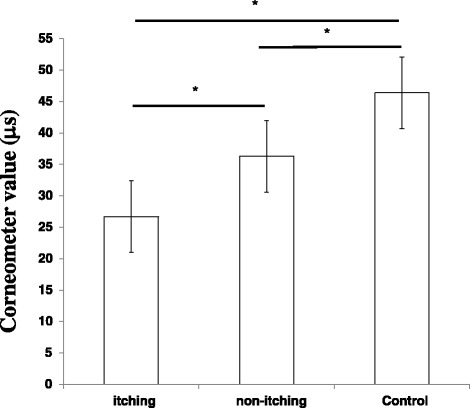
Hydration level of the skin in patients with varicose veins. Values represent the mean ± standard deviation. Dunnett’s method was used to compare between patients with itching and the control group, or between patients without itching and the control group. Tukey-Kramer method was used to compare between patients with itching and those without. *p < 0.05

First, we investigated whether there was a difference in the level of stratum corneum water content between patients with or without itching symptom. The water content of the stratum corneum in patients experiencing itching (26.7 ± 10.6 μs) was significantly lower than that in patients without itching (36.3 ± 10.6 μs) (*p* < 0.05). Moreover, these values were significantly lower compared to the values measured in the non-patient control group (48.8 ± 8.4 μs) (*p* < 0.05) (Fig. 1).

**Fig. 2:**
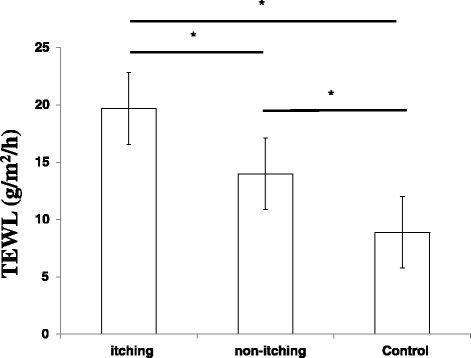
TEWL levels on skin in patients with or without itching. Values represent the mean ± standard deviation. Dunnett’s method was used to compare between patients with itching and the control group, or between patients without itching and the control group. Tukey-Kramer method was used to compare between patients with itching and those without. *p < 0.05

We then compared the level of TEWL in patients with and without itching symptom. TEWL values in patients with itching (19.1 ± 5.2 g/m^2^/h) were significantly higher than that in patients without itching (14.0 ± 6.6 μs) (*p* < 0.05). Moreover, TEWL levels in both patient groups were significantly higher than that in the non-patient control group (8.9 ± 1.6 g/m^2^/h) (*p* < 0.05) (Fig. 2).

**Fig. 3:**
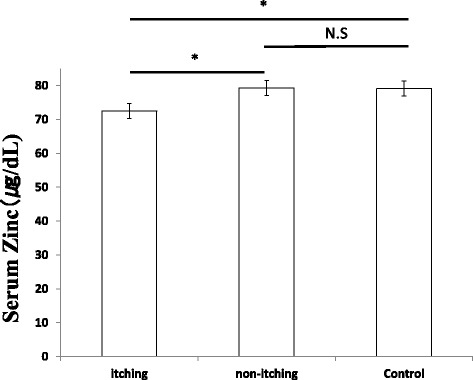
Serum zinc levels in patients with or without itching. Values represent the mean ± standard deviation. Dunnett’s method was used to compare between patients with itching and the control group, or between patients without itching and the control group. Tukey-Kramer method was used to compare between patients with itching and those without. *p < 0.05

**Fig. 4:**
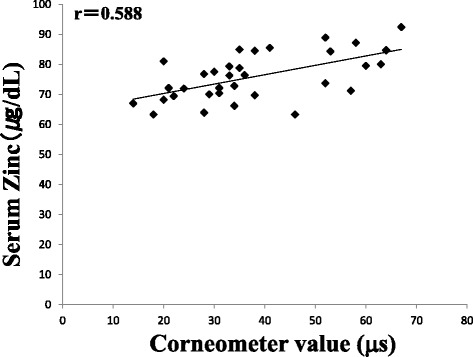
Correlation between the stratum corneum water content and serum zinc level. Relationship between serum zinc and corneal water content is shown by scatter plot and regression line

**Fig. 5:**
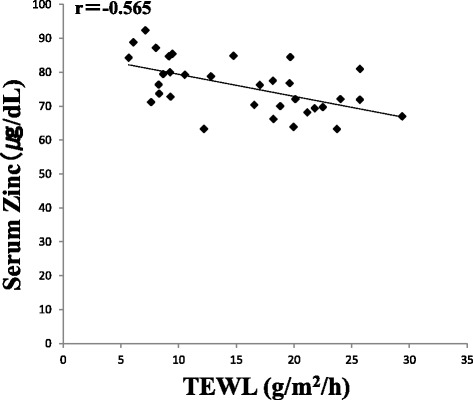
Correlation between TEWL and serum zinc level. Relationship between serum zinc and TEWL is shown by scatter plot and regression line

Next, we measured the serum zinc levels in the study participants. The zinc content was significantly lower in the patients with itching (72.5 ± 7.1 μg/dL) compared to that in the control group (79.1 ± 8.1 μg/dL) (*p* < 0.05). However, no significant difference was observed in the zinc content between patients without itching (79.3 ± 7.4 μg/dL) and the control group (Fig. 3). Additionally, relationships between the stratum corneum water content and TEWL against serum zinc were examined in all participants. There was a positive correlation (*r* = 0.588) between the stratum corneum water content and serum zinc (Fig. 4), and a negative correlation (*r* = − 0.565) was observed between TEWL and serum zinc (Fig. 5).

## Discussion

In this study, we showed the possibility that dry skin is associated with itching experienced by some patients with varicose veins. In addition to this, the stratum corneum water content and TEWL correlated with serum zinc level. These data suggest that serum zinc is affected by the reduction of skin barrier function.

Employing the measurement of stratum corneum water content and TEWL, we evaluated the hydration state of the skin in the patients. The stratum corneum prevents the entry of foreign substances and moisture evaporation from the skin. TEWL measurement is used to determine the amount of moisture evaporating from the stratum corneum and TEWL values reflect the barrier function of the stratum corneum. In general, TEWL values are high in skin diseases such as atopic dermatitis in which the barrier function of skin is impaired [15].

Our results showed that the water content of the stratum corneum in patients with varicose veins was significantly lower compared to that in the non-patient controls, suggesting that these patients have a symptom of dry skin. Patients experiencing itching may suffer from severe dry skin since the average strataum corneum water content in this group of patients was particularly low. The TEWL level in patients with itching was significantly higher than that in patients who are not experiencing itching, and the TEWL level in patients with varicose veins was significantly higher than that in the control group. Together, our results suggest that the barrier function of the skin is decreased in patients with varicose veins who suffer from itching in the affected area.

We then investigated a link between serum concentration of zinc and itch caused by varicose veins. In recent years, it has been considered that zinc deficiency contributes to various skin conditions such as xerosis and acrodermatitis enteropathica [16, 17]. There is a report that zinc deficiency affects the skin barrier and the immune system and is considered a factor that exacerbates atopic dermatitis [18]. In addition, decrease in serum zinc and increase in histamine levels were observed in dialysis patients experiencing itching, indicating a negative correlation between zinc and histamine levels in these patients [12]. In our study, decrease in serum zinc level was observed in patients with itching compared to patients without itching and the control group. No significant difference was seen between patients without itching symptom and non-patient controls. Moreover, histamine level in the patients with varicose veins in the lower extremities was increased compared to healthy individuals (Data not shown).

## Conclusions

Our study showed for the first time that lowered serum zinc level is one of the factors inducing itch caused by varicose veins, and we suggest that zinc supplementation may be an effective treatment to alleviate itching and improve the quality of life in these patients. Our finding could provide a basis for future investigations to elucidate the mechanism(s) of itch due to varicose veins.

### Limitation

The age and sex of the three groups in this study were not matched. It was particularly difficult to collect age-matched controls (healthy individuals). In addition, varicose veins occur more frequently in females, and there were much fewer male patients available for this study.
